# Complex Chronic Wound Biofilms Are Inhibited *in vitro* by the Natural Extract of *Capparis spinose*

**DOI:** 10.3389/fmicb.2022.832919

**Published:** 2022-04-11

**Authors:** Silvia Di Lodovico, Tiziana Bacchetti, Simonetta D’Ercole, Sara Covone, Morena Petrini, Mara Di Giulio, Paola Di Fermo, Firas Diban, Gianna Ferretti, Luigina Cellini

**Affiliations:** ^1^Department of Pharmacy, University “G. d’Annunzio” Chieti-Pescara, Chieti, Italy; ^2^Department of Life and Environmental Sciences, Polytechnic University of Marche, Ancona, Italy; ^3^Department of Medical, Oral and Biotechnological Sciences, University “G. d’Annunzio” Chieti-Pescara, Chieti, Italy; ^4^Department of Clinical Science, Research Center of Health Education and Health Promotion, Polytechnic University of Marche, Ancona, Italy

**Keywords:** *Capparis spinose*, antimicrobial and antivirulence actions, dual-species biofilm, Lubbock chronic wound biofilm model, *S. aureus*, *P. aeruginosa*, *C. albicans*

## Abstract

Resistant wound microorganisms are becoming an extremely serious challenge in the process of treating infected chronic wounds, leading to impaired healing. Thus, additional approaches should be taken into consideration to improve the healing process. The use of natural extracts can represent a valid alternative to treat/control the microbial infections in wounds. This study investigates the antimicrobial/antivirulence effects of *Capparis spinose* aqueous extract against the main chronic wound pathogens: *Staphylococcus aureus*, *Pseudomonas aeruginosa*, and *Candida albicans.* The extract shows phenolic characterization with rutin (1.8 ± 0.14 μg/mg) as the major compound and antibacterial effect against bacteria (*S. aureus* PECHA 10 MIC 6.25%; *P. aeruginosa* PECHA 4 MIC 12.50%) without action against *C. albicans* (MIC and MFC ≥ 50%). *Capparis spinose* also shows a significant antivirulence effect in terms of antimotility/antibiofilm actions. In particular, the extract acts (i) on *P. aeruginosa* both increasing its swimming and swarming motility favoring the planktonic phenotype and reducing its adhesive capability, (ii) on *S. aureus* and *P. aeruginosa* biofilm formation reducing both the biomass and CFU/ml. Furthermore, the extract significantly displays the reduction of a dual-species *S. aureus* and *P. aeruginosa* Lubbock chronic wound biofilm, a complex model that mimics the realistic *in vivo* microbial spatial distribution in wounds. The results suggest that *C. spinose* aqueous extract could represent an innovative eco-friendly strategy to prevent/control the wound microbial infection.

## Introduction

The increase and rapid spread of antimicrobial-resistant wound microorganisms hinder the management of microbial infections and delays wound healing. The wound microbial colonization of wound is the most frequent poly-microbial colonization, involving both aerobic and anaerobic pathogen microorganisms including bacteria and yeasts. Among the detected pathogens, *Staphylococcus aureus*, *Pseudomonas aeruginosa*, *Candida albicans*, and beta-hemolytic streptococci are the primary cause of delayed wound healing and infection (Bowler et al., 2001). In particular, *S. aureus* and *P. aeruginosa* are the main wound bacterial isolates playing an important role in the development of poly-microbial biofilms (DeLeon et al., 2014; Di Giulio et al., 2020).

*Staphylococcus aureus* is the most problematic bacterium in wound infections (Tong et al., 2015) with a high incidence affecting the management practices. As reported by Nakamura et al. (2014), the blood stream infection by *S. aureus* is an important risk factor of wound infection. *Staphylococcus aureus* is able to express various virulence factors that facilitate cell adhesion and host response. In fact, the microorganism binds fibronectin, collagen, fibrinogen, laminin, and elastin, and thanks to its coagulase activity, it produces a fibrin network that represents a scaffold on which bacteria can adhere forming a biofilm (Arciola et al., 2005; Yung et al., 2021). Alves et al. (2018) demonstrated that *S. aureus* acts as a pioneer for the attachment of *P. aeruginosa* that, in turn, promotes an invasive phenotype in *S. aureus*.

*Pseudomonas aeruginosa* is another important pathogen responsible for infections that are difficult to treat including skin diseases. If the wound is not properly treated, *P. aeruginosa* is able to change the infection type from local to systemic. The increase in multidrug -resistant *P. aeruginosa* strains, together with their capability to form a biofilm, represents a challenge for the treatment (Yang et al., 2019). In addition, the dynamic relationship among swimming, swarming, and twitching motility represents a significant virulence bacterium trait, interfering with the biofilm formation (Zolfaghar et al., 2003). Finally, in wound biofilms, also *C. albicans* provides a synergistic microbial complex with bacteria (Di Giulio et al., 2018).

Chronic wound infections are persistent and very hard to eradicate due to the poly-microbial biofilm and the increasing resistant/tolerant microorganisms against traditional treatments. Orazi and O’Toole (2017) reported that in a poly-microbial biofilm, exoproducts of *P. aeruginosa* reduce the susceptibility of *S. aureus* to vancomycin and tobramycin, and the release of N-acetyl glucosamine (GlcNAc) by *S. aureus* stimulates the *P. aeruginosa* quinolone signal (PQS) that is responsible for the production of its virulence factors (e.g., pyocyanin, elastase, rhamnolipids, and HQNO) and quorum sensing. The increase in multidrug-resistant wound strains today represents an important worldwide challenge and new treatment strategies are urgent. New approaches have been developed to act interfering with the ability of the bacteria to produce virulence factors, such as the factors produced during growth as biofilms, which promote resistance to common drugs.

In this scenario, natural compounds could represent innovative approaches, as adjuvants and alternatives to antibiotics in the drainage or debridement to remove sloughed and devitalized tissues, which cause slow wound healing (Di Giulio et al., 2020).

Different natural compounds are proposed to treat microbial proliferation in wounds. Bioactive and antimicrobial properties of extracts of plant phenolic compounds against human pathogens have been widely studied to characterize and develop new medical and pharmaceutical products (Tungmunnithum et al., 2018). In particular, the phenolic fraction of natural compounds is responsible for the organoleptic and biological effects, such as antimicrobial and antibiofilm effects (Di Lodovico et al., 2020b).

Among the different medicinal plants, *Capparis spinose* deserves to be better investigated for its biological properties. *Capparis spinose* (*C. spinosa*), belonging to the *Capparaeae* family, is widely found in the Mediterranean area (especially in France, Spain, Italy, and Algeria) (Al-Snafi, 2015; Rahimi et al., 2020). It is a perennial spiny bush that bears rounded, fleshy leaves and big white to pinkish-white flowers. *Capparis spinose* has been used as a traditional herbal remedy since ancient times for its beneficial effects on human diseases, such as splenomegaly, mental disorders, tubercular glands, rheumatoid arthritis, and gut and skin disease. Mahboubi and Mahboubi (2014) showed that the aqueous extracts from the roots of *C. spinose* display a remarkable antimicrobial activity against *Staphylococcus* spp., *Escherichia coli*, *Helicobacter pylori*, *Candida* spp., and *Aspergillus niger*.

The aim of this study is to investigate the antimicrobial and antivirulence effects of *C. spinose* aqueous extract against the main chronic wound pathogens. The antivirulence analysis is performed by evaluating *C. spinose* antimotility/twitching and antibiofilm actions. The poly-microbial biofilm is also analyzed by using the Lubbock chronic wound biofilm (LCWB) model that mimics the realistic microbial spatial proliferation in wounds. This *in vitro* model is widely recognized as more closely resembling the *in vivo* human wound environment including the wound-simulating medium, the fibrin network produced by *S. aureus*, and the realistic nutrient and oxygen gradient (Thaarup and Bjarnsholt, 2021). This suitable *in vitro* model represents a pivotal preliminary screen useful in translating into *in vivo* detections.

The proposed study can be defined as “green research” in line with the identification of novel strategies to overcome antimicrobial resistance and for the low environmental impact in the aqueous extraction that is widely diffused in the Mediterranean area.

The innovative aspect of this work is to propose a valid and eco-friendly non-antibiotic strategy to prevent and control wound microbial infections, strongly highlighting the antimicrobial and antivirulence actions of *C. spinose* aqueous extract.

## Materials and Methods

### Bacterial Cultures

Anonymized clinical *Staphylococcus aureus* PECHA 10, *Pseudomonas aeruginosa* PECHA 4, and *Candida albicans* X3 strains (Di Giulio et al., 2018, 2020) were used for this study. The strains were isolated from chronic wounds of patients that gave their informed consent for the study. The study was approved by the Inter Institutional Ethic Committee of University “G. d’Annunzio” Chieti-Pescara, Chieti, Italy (ID n. richycnvw). The strains were characterized for their susceptibility to antibiotics, and in particular, *S. aureus* PECHA 10 and *P. aeruginosa* PECHA 4 were resistant strains (Supplementary Table 1). All the methods were performed in accordance with the relevant guidelines and regulations. For the experiments, bacteria were cultured in Trypticase Soy Broth (TSB, Oxoid, Milan, Italy) and incubated at 37°C overnight in aerobic condition and then refreshed for 2 h at 37°C in an orbital shaker. Then the broth cultures were standardized to optical density at 600 nm (OD_600_) = 0.125. The broth culture of *Candida albicans*, grown on Sabouraud dextrose agar (SAB, Oxoid, Milan, Italy) was prepared in RPMI 1640 (Sigma-Aldrich, Milan, Italy) plus 2% glucose and standardized to OD_600_ = 0.15.

### Chemicals

Gallic acid, catechin, chlorogenic acid, p-OH benzoic acid, vanillic acid, epicatechin, syringic acid, 3-OH benzoic acid, 3-OH-4-MeO benzaldehyde, p-coumaric acid, rutin, sinapinic acid, t-ferullic acid, naringin, 2,3-diMeO benzoic acid, benzoic acid, o-coumaric acid, quercetin, harpagoside, t-cinnamic acid, naringenin, and carvacrol were purchased from Sigma-Aldrich (Milan, Italy). Methanol (HPLC-grade) and formic acid (99%) were obtained from Carlo Erba Reagenti (Milan, Italy).

### Extract Preparation

Plants of *C. spinose* subsp. *rupestris* have been growing in Borgo Cisterna (Santa Lucia Cisterna, Macerata Feltria, PU, Italy) and managed by the Agency for Food Service Industry in the Marche (ASSAM), an institution involved in the implementation of programs for the protection of biodiversity for agriculture of the Marche Region in relation to the Regional Law No. 12 “Protection of animal and plant genetic resources of the Marche” approved June 2003. The law protects the genetic resources that are locally grown within the region. The flower bods used in this study have been kindly provided by Mario Gallarani and his family that grow *C. spinose* subsp. *rupestris* in Borgo Cisterna (Santa Lucia Cisterna, Macerata Feltria, Italy). Plants of *C. spinose* subsp. rupestris have been registered to the Regional Register of Biodiversity of Marche Region No. 70 of the Vegetal Section, Herbaceous Species. The use of *C. spinose* was in agreement with the IUCN Policy Statement on Research Involving Species at Risk of Extinction and the Convention on the Trade in Endangered Species of Wild Fauna and Flora. *Capparis spinose* flower bods were washed, frozen at –20°C, freeze-dried, and shredded. One gram of the powdered sample was incubated with 100 ml of ultrafiltered water at 80°C for 10 min (Eddouks et al., 2017). Thereafter, the aqueous extract was filtered by Millipore filter (Millipore 0.2 mm) to remove particulate matter.

### High-Performance Liquid Chromatography Analyses

High-performance liquid chromatography (HPLC) **analyses** were performed on Waters liquid chromatograph equipped with a model 600 solvent pump and a 2996 photodiode array detector, and Empower v.2 Software (Waters Spa, Milford, MA, United States) was used for acquisition of data. C18 reversed-phase packing column [Prodigy ODS(3), 4.6 × 150 mm, 5 μm; Phenomenex, Torrance, CA, United States] was used for separation, and the column was thermostated at 30 ± 1°C using a Jetstream2 Plus column oven. The UV/Vis acquisition wavelength was set in the range of 200–500 nm. The quantitative analyses were achieved at maximum wavelength for each compound. The injection volume was 20 μl. The mobile phase was directly on-line degassed by using Biotech DEGASi, mod. Compact (LabService, Anzola dell’Emilia, Italy). Gradient elution was performed using the mobile phase water–acetonitrile (93:7, v/v, 3% acetic acid) (Zengin et al., 2017). The sample solutions were centrifuged, and the supernatant was injected into HPLC.

The phenolic stock solutions were prepared at a concentration of 1 mg/ml in a final volume of 10 ml of methanol. Working solutions of mixed standards at different concentrations obtained by dilution in mobile phase were injected into the HPLC-UV/Vis system.

The lyophilized extract sample was weighted and dissolved in mobile phase, and 20 μl was injected into the HPLC-UV/Vis system. For over range samples, 1:10 dilution factor was applied.

### Assessment of Total Phenolic and Flavonoid Content and Antioxidant Activity

Total phenolic (TP) content in *C. spinose* has been evaluated by Folin–Ciocalteu assay (Ainsworth and Gillespie, 2007). Total flavonoid (TF) content in *C. spinose* was measured by spectrophotometry with aluminum chloride (AlCl_3_) as the reagent according to Kim et al. (2003). TP and TF levels were expressed as milligrams of gallic acid equivalent (GAE) per 100 g of dry weight of *C. spinose* (mg GAE/100 gdw) and milligrams of catechin equivalent (CE) per 100 g of dry weight of *C. spinose* (mg CE/100 gdw), respectively.

Total antioxidant capacity (TAC) of *C. spinose* was determined by oxygen radical absorbance capacity (ORAC) assay, using fluorescein, as fluorescent probe, and 2,2’-azobis (2-methylpropionamide) dihydrochloride (AAPH), as oxidizing agent (Gillespie et al., 2007). Trolox was used to calibrate the assay. The final ORAC values were calculated using the net area under the curve (AUC) of decay. Results were expressed as Trolox equivalents per 100 g of dry *C. spinose* weight (mmol TE/100 gdw).

### *Capparis spinose* Aqueous Extract Antimicrobial Assays

The *C. spinose* aqueous extract MIC was performed against *S. aureus* PECHA 10, *P. aeruginosa* PECHA 4, and *C. albicans* X3 by microdilution method according to the CLSI (2018). *Capparis spinose* aqueous extract stock solution was diluted in Mueller Hinton Broth II cation adjusted (MHB, Oxoid, Milan, Italy) for bacteria and in RPMI 1640 plus 2% glucose for *C. albicans* X3 at a final concentration of from 50 to 0.78%. MBCs/MFCs were determined by subculturing 10 μl of suspensions from the MICs on Mueller Hinton agar (MHA, OXOID, Milan, Italy) for bacteria and on Sabouraud Dextrose agar (SAB, OXOID, Milan, Italy) for *C. albicans*.

As control, amikacin and amphotericin B MICs were used for bacteria and *C. albicans* X3, respectively.

### *Capparis spinose* Aqueous Extract Antivirulence Assays

The antivirulence analysis of *C. spinose* aqueous extract was performed by evaluating its effect on *P. aeruginosa* PECHA 4 motility (swimming and swarming), *P. aeruginosa* PECHA 4 twitching, *S. aureus* PECHA 10, and *P. aeruginosa* PECHA 4 biofilm formation.

#### Effect on *Pseudomonas aeruginosa* PECHA 4 Motility

The *C. spinose* aqueous extract capability to interfere with the *P. aeruginosa* PECHA 4 motility was determined by swarming and swimming motility. Briefly, according to Abraham et al. (2011), for the swarming motility, the standardized cultures were inoculated at the center of swarming plates containing 1% peptone, 0.5% NaCl, 0.5% agar, and 0.5% D-glucose with the extract at sub-MICs. For swimming motility, standardized cultures were inoculated at the center of plates containing 1% tryptone, 0.5% NaCl, and 0.3% agar and extract at sub-MICs. Plates were incubated at 37°C for 24 h, and bacterial halos were recorded.

#### Effect on *Pseudomonas aeruginosa* PECHA 4 Twitching

The capability of *C. spinose* aqueous extract to interfere with the *P. aeruginosa* PECHA 4 pilus retraction was determined by twitching assay. For the cultural analysis, cultures were inoculated to the bottom of the twitching plates consisting of 10 g/L of tryptone, 5 g/L of yeast extract, 10 g/L of NaCl, and 1% agar with the extract at sub-MICs. Plates were incubated at 37°C for 24 h, and then the agar was removed, and the halo was stained with 0.1% Crystal Violet and measured (Turnbull and Whitchurch, 2014). For RT-PCR *pilT* gene expression, *P. aeruginosa* PECHA 4 RNA was extracted using the RNeasy mini kit (Qiagen, Milan, Italy), according to the manufacturer’s instructions. cDNA was generated using the iScript cDNA Synthesis Kit (Bio-Rad, Milan, Italy) and then stored at –20°C until use. For the quantitative PCR, the oligonucleotide used primers as follows: *pilT* Fwd: ACCGACTTCTCCTTCGAGGT; *pilT* Rev: GAGGGAATGGTCCGGAATAC (Cowles and Gitai, 2010); the housekeeping gene 5S RNA Fwd: TGACGATCATAGAGCGTTGG; 5S RNA Rev: GATAGGAGCTTGACGATGACCT (El-Sayed et al., 2020). Quantitative PCR reactions were performed according to Di Fermo et al. (2020). A melting curve was used at the end to confirm only one peak and only one product. Values of the threshold cycle (Ct) and relative expression level were normalized by the ΔCT method. Results were analyzed using the Bio-Rad CFX Manager Software, version 3.1 (Bio-Rad Laboratorie, Milan, Italy).

#### Effect on *Staphylococcus aureus* PECHA 10 and *Pseudomonas aeruginosa* PECHA 4 Biofilm Formation

Considering the ineffectiveness of *C. spinose* against *C. albicans* X3, the antibiofilm effect was not detected. *Capparis spinose* aqueous extract antibiofilm effect at sub-MICs was evaluated on *S. aureus* PECHA 10 and *P. aeruginosa* PECHA 4 biofilm formation in terms of (i) biomass quantification, (ii) CFU/ml, and (iii) cell viability.

For biomass quantification, standardized cultures in TSB (Oxoid) plus 0.5% (vol/vol) glucose were inoculated in 96-well flat-bottom microtiter plates in the presence of 1/2, 1/4, 1/8 MIC, or without (control) the extract. Plates were incubated at 37°C for 24 h in aerobic condition. After incubation, dry biofilms were stained with 0.1% Crystal Violet and quantified according to Di Lodovico et al. (2020b).

For CFU/ml determination, after 24 h, each well was washed with PBS, and adhered bacteria were scraped off and resuspended in 200 μl of PBS, transferred to test tubes, vortexed for 2 min, diluted, and spread on mannitol salt (MSA, OXOID, Milan, Italy) agar for *S. aureus* PECHA 10 and on cetrimide (CET, OXOID, Milan, Italy) for *P. aeruginosa* PECHA 4. Plates were incubated for 24 h at 37°C (D’Ercole et al., 2020). Microscopic observations with Live/Dead staining prior to spreading confirmed the presence of disaggregated viable cells.

Cell viability was evaluated by using Live/Dead staining (Molecular Probes Inc., Invitrogen, San Giuliano Milanese, Italy) according to Turnbull and Whitchurch (2014) and Di Lodovico et al. (2019). The number of viable and dead cells was determined by using an image analysis software (LEICA QWin) through the examination of at least 10 random fields of view, and the counts were repeated independently by three blinded microbiologists (Di Lodovico et al., 2020a).

#### Effect on Dual-Species Biofilm, Lubbock Chronic Wound Biofilm

To evaluate the effect of *C. spinose* aqueous extract in a dual-species *S. aureus* and *P. aeruginosa* biofilm, in-forming Lubbock chronic wound biofilm (LCWB) was prepared according to Di Giulio et al. (2020). Briefly, 100 μl of extract at a final concentration of 3.12%, which corresponded to 1/2 MIC of *S. aureus* PECHA 10, or 100 μl of amikacin (AMK, as a reference) at a final concentration of 8 mg/L or 100 μl of PBS (for control), was added to the Lubbock medium containing Brucella broth (BB, Oxoid, Milan, Italy) with 0.1% agar bacteriological, 50% porcine plasma (Sigma Aldrich, Milan, Italy), 5% horse erythrocytes (BBL, Microbiology System, Milan, Italy), 2% fetal calf serum (Biolife Italiana, Milan, Italy), and *S. aureus* and *P. aeruginosa* grown in TSB (Oxoid) (Di Giulio et al., 2020). The test tubes were incubated for 48 h at 37°C, and then the *S. aureus* PECHA 10 and *P. aeruginosa* PECHA 4 CFUs per mg of LCWB were determined (Di Giulio et al., 2020).

### Statistical Analysis

Data were obtained from at least three independent experiments performed in duplicate. Data were shown as the means ± standard deviation. Differences between groups were assessed with one-way analysis of variance (ANOVA). Values of *p* ≤ 0.05 were considered statistically significant.

## Results

This study evaluates the antimicrobial and antivirulence properties of a well-characterized *C. spinose* aqueous extract against microorganisms isolated from chronic wounds.

### High-Performance Liquid Chromatography Analysis

Only quantifiable phenolic compounds greater than the limit of quantification (LOQ = 0.20 μg/ml) are shown in the Table 1. All other compounds are to be understood as not detected or below the detection limit (LOD = 0.10 μg/ml).

**TABLE 1:** Total amounts (μg/mg) of phenolics in the lyophilized extract.

	Concentration (μg/mg)
Chlorogenic acid	0.31 ± 0.04
*p*-OH benzoic acid	0.35 ± 0.07
3-OH benzoic acid	0.40 ± 0.05
*p-*Coumaric acid	0.54 ± 0.03
Rutin	1.83 ± 0.14
2,3-diMeO benzoic acid	0.46 ± 0.04
Total	3.89 ± 0.18

### Assessment of Total Phenolic and Flavonoid Content and Antioxidant Activity

Total phenolic (TP) and total flavonoid (TF) levels in *C. spinose* aqueous extract are 1.6 ± 0.1 g GAE/100 g and 91.1 ± 19.3 mg CE/100 g, respectively. Total antioxidant capacity, evaluated by ORAC assay, is 50.8 ± 8.4 mmol TE/100 g.

### *Capparis spinose* Aqueous Extract Antimicrobial Assays

Table 2 shows the MIC and the MBC/MFC values of *C. spinose* aqueous extract against *S. aureus*, *P. aeruginosa*, and *C. albicans* clinical isolates.

**TABLE 2:** Minimum inhibitory concentration (MIC) and minimum bactericidal concentration (MBC)/minimum fungicidal concentration (MFC) of *Capparis spinose* aqueous extract against *Staphylococcus aureus* PECHA 10, *Pseudomonas aeruginosa* PECHA, and *Candida albicans* X3.

Strains	MIC		MBC/MFC	
	*C. spinose* aqueous extract (%)	Amikacin/amphotericin B (μg/ml)	*C. spinose* aqueous extract (%)	Amikacin/amphotericin B (μg/ml)
*S. aureus* PECHA 10	6.25	16	12.50	16
*P. aeruginosa* PECHA 4	12.50	32	25	32
*C. albicans* X3	>50	0.5	>50	0.5

*Amikacin and amphotericin B are included as control for bacteria and C. albicans X3, respectively.*

According to *C. spinose* aqueous extract gallic acid and catechin equivalent analysis, the *S. aureus* MIC corresponds to 0.78 mg GAE/ml and 0.11 mg CE/ml, and the MBC corresponds to 1.56 mg GAE/ml and 0.22 mg CE/ml. The *P. aeruginosa* MIC corresponds to 1.56 mg GAE/ml and 0.22 mg CE/ml, and the MBC corresponds to 3.12 mg GAE/ml and 0.44 mg CE/ml. For *C. albicans*, the MIC and the MFC are more than 6.25 mg GAE/ml and 0.90 mg CE/ml.

*Capparis spinose* aqueous extract shows a relevant antibacterial effect against the tested bacterial strains.

Considering the ineffectiveness of *C. spinose* aqueous extract against *C. albicans* (MIC and MFC values are more than 50%, that is, the maximum percentage of the extract tested in this study), this microorganism is not included in the subsequent experiments.

### *Capparis spinose* Aqueous Extract Antivirulence Assays

#### Effect on *Pseudomonas aeruginosa* PECHA 4 Motility

**FIGURE 1:**
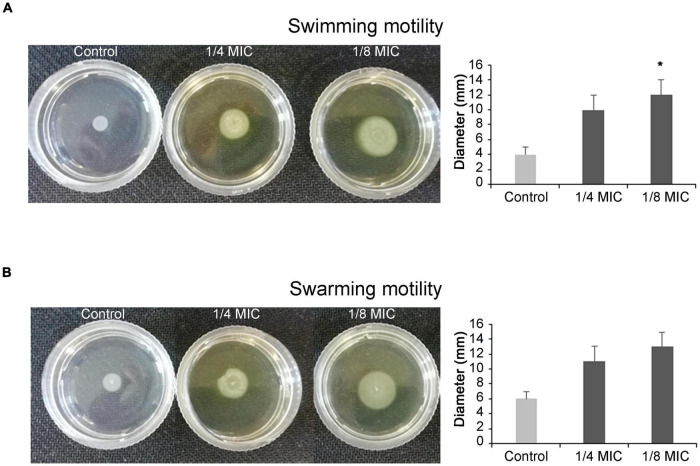
Effect of sub-minimum inhibitory concentrations (MICs) of *Capparis spinose* aqueous extract on *Pseudomonas aeruginosa* PECHA 4 motility assay; representative images of swimming **(A)** and swarming **(B)** motilities on soft agar plates with diameter of obtained halos and relative histograms. *Significant with respect to the control (*p* < 0.05).

The *C. spinose* aqueous extract displays a significant increase in the swimming and swarming motility of *P. aeruginosa*. As shown in Figures 1A,B, the swimming and swarming halo sizes are more than the control ones. In fact, the halo diameter of swimming motility for the control is 4 ± 1 mm, whereas 10 ± 2 and 12 ± 2 mm are halo diameters recorded in the presence of 1/4 and 1/8 MIC of *C. spinose* aqueous extract, respectively. The halo diameters for swarming motility are 6 ± 1 mm for control, 11 ± 3 and 13 ± 3 mm for 1/4 and 1/8 MIC of *C. spinose* aqueous extract, respectively.

*Capparis spinose* aqueous extract enhances *P. aeruginosa* flagellar activation.

#### Effect on *Pseudomonas aeruginosa* PECHA 4 Twitching

**FIGURE 2:**
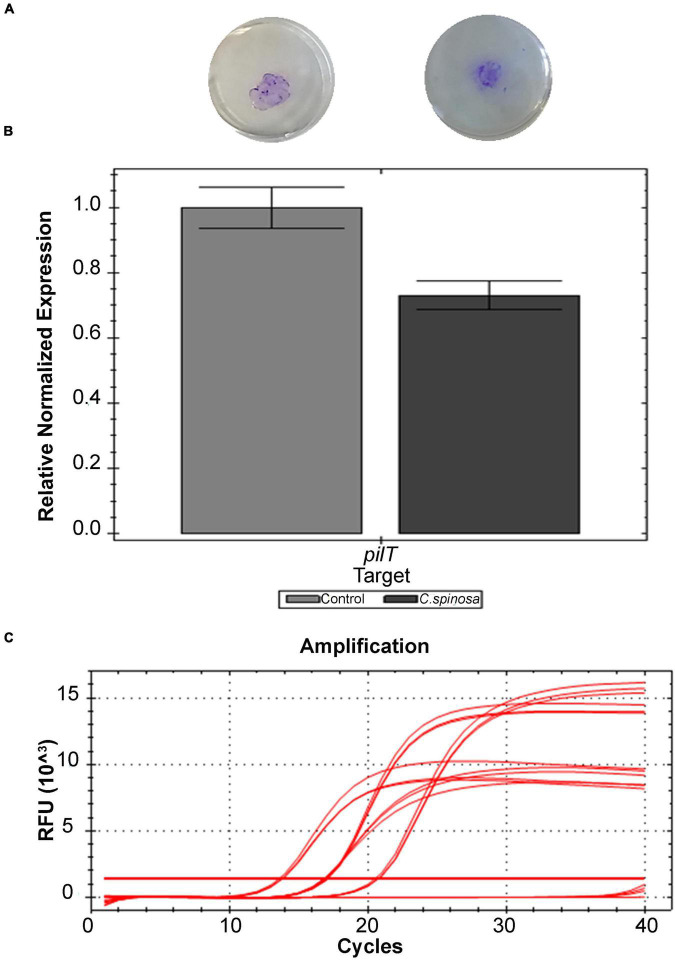
Effect of sub-MICs of *Capparis spinose* aqueous extract on *Pseudomonas aeruginosa* PECHA 4 twitching assay: **(A)** representative images of macroscopic twitching assay on cultural plates; **(B)** expression of *pilT* gene; **(C)** amplification chart with the Ct values of each sample of the *pilT* gene expression.

For twitching motility, relevant percentages of halo reduction with respect to the control are obtained. As shown in Figure 2A, the halo diameters are 12 ± 4 mm for the control and 9 ± 1 mm in the presence of both 1/4 and 1/8 MIC of *C. spinose* aqueous extract with 23% ± 15% of halo reduction. These data are also confirmed by RT-PCR that evaluates the *pilT* gene expression. *Capparis spinose* aqueous extract at sub-MIC concentrations reduces the *pilT* expression of 27% (Figures 2B,C).

*Capparis spinose* aqueous extract interferes with the *P. aeruginosa* pilus retraction, reducing the microorganism adhesive capability.

#### Effect on *Staphylococcus aureus* PECHA 10 and *Pseudomonas aeruginosa* PECHA 4 Biofilm Formation

**FIGURE 3:**
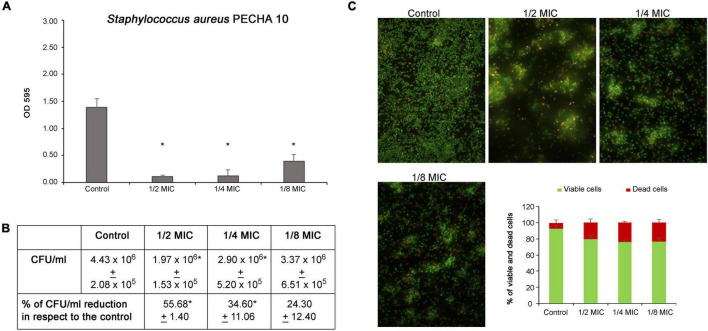
Effect of sub-MICs of *Capparis spinose* aqueous extract on *Staphylococcus aureus* PECHA 10 biofilm formation. **(A)** Biomass evaluation; **(B)** cultivable cell count (CFU/ml); **(C)** representative images (Live/Dead stain) and quantitative analysis (histograms) of viable and dead cells. *Significant with respect to the control (*p* < 0.05).

The *C. spinose* aqueous extract shows a significant action in antibiofilm formation against both *S. aureus* and *P. aeruginosa* detected strains. As shown in Figure 3A, the *C. spinose* aqueous extract, at sub-MIC values, significantly reduces the *S. aureus* biomass after 24 h of treatment. In particular, the percentages of biomass reduction with respect to the control are 92.21% ± 1.87, 90.54% ± 8.38, and 72.63% ± 6.62 at 1/2, 1/4, and 1/8 MICs, respectively. Figure 3B shows the *S. aureus* CFU/ml of biofilm formation after treatment with sub-MICs of *C. spinose* aqueous extract for 24 h. With respect to the control, there is a significant CFU/ml reduction (*p* < 0.05) at 1/2 and 1/4 MICs. In the presence of *C. spinose* aqueous extract at sub-MICs, there is a relevant reduction in biofilm adhesion with remarkable red cells detected in Live/Dead images (Figure 3C). The cells appear less clustered with about 20% of dead red cells (Figure 3C, histograms).

**FIGURE 4:**
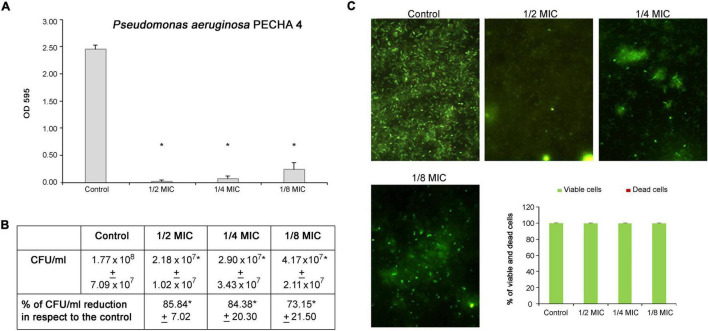
Effect of sub-MICs of *Capparis spinose* aqueous extract on *Pseudomonas aeruginosa* PECHA 4 biofilm formation. **(A)** Biomass evaluation; **(B)** cultivable cell count (CFU/ml); **(C)** representative images (Live/Dead stain) and quantitative analysis (histograms) of viable and dead cells. *Significant with respect to the control (*p* < 0.05).

Regarding the *P. aeruginosa* antibiofilm formation activity, significant percentages of biomass reduction are obtained at sub-MICs. In detail, the percentages range from 89.97% ± 4.97 at 1/8 MIC to 99.48% ± 0.76 at 1/2 MIC (Figure 4A). In the presence of *C. spinose* aqueous extract, significant reductions (*p* < 0.05) in biomass and CFU/ml are observed. In fact, a few cells are detected by CFU enumeration (Figure 4B) and Live/Dead staining (Figure 4C). This significant *P. aeruginosa* reduction in the presence of *C. spinose* aqueous extract is confirmed by Live/Dead images (Figure 4C) with an almost total percentage of viable cells (Figure 4C, histograms).

*Capparis spinose* aqueous extract is able to inhibit the biofilm formation of *S. aureus* and *P. aeruginosa* tested strains.

#### Effect on Dual-Species Biofilm, Lubbock Chronic Wound Biofilm

In a poly-microbial biofilm, *C. spinose* aqueous extract significantly reduces the microbial growth. In fact, in dual-species *S. aureus* and *P. aeruginosa* LCWB, the CFU/mg reductions are 97.32% ± 2.29 (*p* < 0.05) for *S. aureus* and 99.67% ± 0.07 (*p* < 0.05) for *P. aeruginosa* (Table 3). This relevant reduction is similar to those obtained with the antibiotic used as reference control.

**TABLE 3:** Colony-forming units (CFUs)/mg of *Staphylococcus aureus* PECHA 10 and *Pseudomonas aeruginosa* PECHA 4 in the presence of *Capparis spinose* aqueous extract in informing Lubbock chronic wound biofilm (LCWB) model [amikacin (AMK) as reference control].

	CFUs/mg LCWB (% reduction with respect to the control)	
	*S. aureus* PECHA 10	*P. aeruginosa* PECHA 4
***C. spinose***	7.03 × 10^1*^ ± 6.00 × 10^1^ (97.32* ± 2.26)	1.66 × 10^3*^ ± 3.55 × 10^2^ (99.67* ± 0.07)
**AMK**	2.56 × 10^2^ ± 1.03 × 10^2^ (90.20* ± 1.53)	2.32 × 10^4^ ± 5.88 × 10^3^ (95.40* ± 1.17)
**Control**	2.62 × 10^3^ ± 5.37 × 10^2^	5.03 × 10^5^ ± 3.66 × 10^5^

*Statistically significant with respect to the control (p < 0.05).

*Capparis spinose* aqueous extract confirms its antibiofilm effect in the complex system LCWB model with a significant capability to reduce the microbial growth.

## Discussion

In this study, the antimicrobial and antivirulence activities of a characterized *C. spinose* aqueous extract have been evaluated against resistant chronic wound microorganisms. The worrying phenomenon related to the antimicrobial resistance of chronic wound microorganisms is the cause of treatments failure. In addition, the chronic wound infections are always associated with the poly-microbial biofilm delaying wound healing (Di Giulio et al., 2020).

The World Health Organization reports the use of different medicinal plants for the management of health and treatment of diseases due to their bioactive components and health-promoting effects (World Health Organization (WHO), 2013).

Caper berries contain a wide range of bioactive compounds, such as alkaloids, flavonoids, steroids, terpenoids, and tocopherols. Healthy properties and composition of phytonutrients of Capparis flower buds have been recently reviewed. Levels and composition of phytonutrients are influenced by different factors such as cultivars, genotypes (both cultivated and wild), and geographical origin (Maldini et al., 2016; Zhang and Ma, 2018; Wojdyło et al., 2019).

The profile of the main phenolic compounds, detected in the present study, is in a greater part in agreement with previous studies (Mollica et al., 2017; Stefanucci et al., 2018) with the exception of the amount of rutin that represents the compound in major amount. It is important to mention that the phenolic profile could change according to the applied extraction technique and the extraction solvent, which is confirmed by several studies in the literature (Mollica et al., 2017, 2019; Stefanucci et al., 2018). In particular, Stefanucci et al. (2018) demonstrated a large variability in rutin concentration in Capparis collected in Italy, Morocco, and Turkey (Mollica et al., 2017, 2019).

The studied *C. spinose* aqueous extract affects the bacterial growth without any impact on yeast cells, showing a selective action against bacteria. In fact, the compound shows a significant antimicrobial action against *S. aureus* and *P. aeruginosa* clinical isolates without a relevant effect against *C. albicans*. The detected MIC values are in the MIC range previously found by Taguri et al. (2006) who determined the MICs of 22 polyphenols against 26 species of bacteria with MIC values between 0.067 and 3.200 g/L.

The molecular mechanisms of antibacterial action of phytochemicals, such as phenolic compounds, are not yet fully understood, but these compounds are known to involve many sites of action at cellular level (Bouarab-Chibane et al., 2019). Several authors explained the antimicrobial activity of polyphenols by modifications in the cell membrane permeability, changes in intracellular functions due to interactions between the phenolic compounds and cell enzymes, or by the modification of the cell wall rigidity with integrity losses due to different interaction polyphenol cell membrane (Ikigai et al., 1993; Stapleton et al., 2004; Taguri et al., 2006; Cushnie and Lamb, 2011). Among polyphenols, the main category included in Capparis extract are anthocyanins. We suggest that the observed antibacterial effect could be related to these molecules and/or the synergisms with other antioxidant polyphenols, such as phenolic acids, and their mixtures of different chemical forms. Moreover, according to the *C. spinose* aqueous extract characterization, the rutin could be responsible for the action against *S. aureus* and *P. aeruginosa* growth interfering with DNA synthesis, an antibacterial mechanism of action of various flavonoids (Cushnie and Lamb, 2005). In addition, as reported by Cushnie and Lamb (2005), the flavonoid toxicity is minimal; in fact, they are widely spread in edible plant and beverages. However, for the *in vivo* application, the evaluation of the eventual toxic effect should be done.

*Capparis spinose* aqueous extract displays also a relevant antivirulence action against *P. aeruginosa* motility/twitching and *S. aureus* and *P. aeruginosa* mono- and poly-microbial biofilms. In fact, the tested extract acts both on swimming/swarming motility favoring the flagellar-mediated movement and twitching motility reducing the *P. aeruginosa* adhesion. The detected significant increase in flagellar biosynthesis, with respect to the control, favors the planktonic status leading to enhance the bacterial movement. This effect induces a phenotype more susceptible to treatments. The noticed microbial twitching reduction interferes with the *P. aeruginosa* adhesive capability. The twitching motility is an important step for bacterial colonization and biofilm formation in *P. aeruginosa* (Shreeram et al., 2018). In particular, the inactivation and loss of function in *pilT* produces a hyperpiliation and the loss of twitching motility due to the inability of pilus fiber formation. The inactivation of *pilT* determines the loss of cytotoxicity *in vitro* and the inhibition of the contact between bacteria and the host cells (Shreeram et al., 2018). Here, *C. spinose* extract acts weakening the *P. aeruginosa* adhesive capability.

The microbial biofilm mode of growth allows microbes to protect themselves against host immune system and antimicrobial agents making biofilm-related infections difficult to treat and eradicate. In this study, *C. spinose* aqueous extract significantly reduces the mono- and poly-microbial biofilms of *S. aureus* and *P. aeruginosa* with less clustered cells. On mono-microbial biofilms, the effect is more relevant against *P. aeruginosa* with a significant effect on biomass quantification and bacterial cells. On *S. aureus* biofilm formation, the compound reduces the biomass production with a slow bacterial growth reduction. Cosa et al. (2019) showed that the antibiofilm effect of *C. spinose* is correlated to its capability to reduce the quorum-sensing (QS) regulation, reducing the bacterial virulence and pathogencity. In fact, Abraham et al. (2011) showed that the *C. spinose* methanolic extract is able to reduce the production of AHL-dependent QS interfering with biofilm production. In addition, the authors demonstrated the capability of the extract to reduce the EPS production in different bacterial pathogens. In particular, Peng et al. (2018) underline the significant role of rutin in the QS regulation with AI-2 decreasing and the reduction of biofilm formation and virulence factor gene expression.

A very interesting result is obtained when the effect of *C. spinose* aqueous extract is detected in a condition of poly-microbial biofilm that reproduces the *in vivo* spatial microbial colonization of *S. aureus* and *P. aeruginosa* in a chronic wound. The used LCWB model represents a recognized *in vitro* chronic wound system for interkingdom microbial species. In this model, the presence of red blood cells, plasma, and nutrients, mimicking the wound bed environment, promotes the *S. aureus*/*P. aeruginosa* microbial growth, closely reproducing their spatial distribution in human-like environment. In this complex dual-species microbial colonization, *C. spinose* aqueous extract expresses a significant percentage of reduction of both microbial populations. This interesting data is obtained with a well-recognized *in vitro* model that resembles to the *in vivo* wound environment in terms of wound-simulating media, host matrix, several chosen species, 3D gradients, flow, grown on solid surface (Thaarup and Bjarnsholt, 2021). As a consequence, our results stimulate further studies on *in vivo* model such as porcine and human models. In fact, the limitation of our model, while considering both the presence of the most important chronic wound factors and the easy reproducibility with ethical sound, is the unshared interaction between the immune system and microorganisms. The complex and dynamic events related to the immune host defense should be taken into account in future studies (de Bont et al., 2019; Sabbatini et al., 2021).

In conclusion, the obtained findings suggest the capability of *C. spinose* aqueous extract to inhibit the growth and virulence of *P. aeruginosa* PECHA 4 and *S. aureus* PECHA 10 chronic wound microorganisms. The significant antimicrobial and antivirulence properties make the *C. spinose* a good candidate for the study of novel medicaments in the prevention and control of chronic wound microorganisms.

*Capparis spinose* aqueous extract could represent a valid eco-friendly suggestion to overcome the worrying antimicrobial resistance phenomenon.

## Data Availability Statement

The raw data supporting the conclusions of this article will be made available by the authors, without undue reservation.

## Author Contributions

SDL, MDG, SDE, PDF and FD conducted the microbiology experiments. SC, TB, and GF performed the *Capparis spinose* characterization. MP and SDL performed the data analysis. SDL and LC wrote the manuscript. MDG, LC, and GF contributed to discussing the results and critical review of the manuscript. All authors made significant contributions to this manuscript, participated actively in the conception and design of the experiments, and read and approved the final manuscript.

## Conflict of Interest

The authors declare that the research was conducted in the absence of any commercial or financial relationships that could be construed as a potential conflict of interest.

## Publisher’s Note

All claims expressed in this article are solely those of the authors and do not necessarily represent those of their affiliated organizations, or those of the publisher, the editors and the reviewers. Any product that may be evaluated in this article, or claim that may be made by its manufacturer, is not guaranteed or endorsed by the publisher.
