# Supplementary material for: Complex Chronic Wound Biofilms Are Inhibited in vitro by the Natural Extract of Capparis spinose
Source: Front Microbiol. 2022 Apr 11;13:832919. doi: 10.3389/fmicb.2022.832919 (PMC9035792; doi:10.3389/fmicb.2022.832919)
Supplement: Supplementary file 1 [file Table_1.DOCX]

Supplementary Material

**Table 1S** Antimicrobial susceptibility panel of *Staphylococcus aureus* PECHA 10*, Pseudomonas aeruginosa* PECHA 4 and *Candida albicans* X3 clinical strains used in this study

|  | ***Staphylococcus aureus***  **PECHA 10** | ***Pseudomonas aeruginosa* PECHA 4** | ***Candida albicans***  **X3** |
| --- | --- | --- | --- |
| Amikacin | R | R | - |
| Amoxicillin | - | R | - |
| Aztreonam | - | R | - |
| Cefotaxime | - | R | - |
| Ceftazidime | - | R | - |
| Ceftriaxone | - | R | - |
| Cefalotin | - | R | - |
| Ciprofloxacin | S | R | - |
| Clindamycin | S | - | - |
| Erythromycin | S | - | - |
| Gentamicin | S | R | - |
| Imipenem | - | S | - |
| Levofloxacin | S | R | - |
| Linezolid | S | - | - |
| Moxifloxacin | S | - | - |
| Nitrofurantoin | S | R | - |
| Oxacillin | S | - | - |
| Penicillin G | S | - | - |
| Piperacillin | - | R | - |
| Rifampin | S | - | - |
| Teicoplanin | S | - | - |
| Tetracycline | S | - | - |
| Tobramicin | - | R | - |
| Vancomycin | S | - | - |
| Amphotericin-B | - | - | S |
| Fluconazole | - | - | S |
| Flucytosine | - | - | S |
| Micafungin | - | - | S |
| Voriconazole | - | - | S |
